# Effects of Different Pre-Sowing Treatments and Soil Substrates on Seed Germination of *Salvia przewalskii* Maxim

**DOI:** 10.3390/plants15131991

**Published:** 2026-06-27

**Authors:** Wen-Ke Ji, Xi-Juan Chen, Hong-Qiang Lin, Jian-Pan Xin, Han-Wen Xiao

**Affiliations:** 1 College of Landscape Architecture, Nanjing Forestry University, Nanjing 210037, China; wenkejiii@outlook.com; 2 Shangqiu Normal University, Shangqiu 476000, China; xjchen2013@163.com; 3 Sichuan Wolong National Nature Reserve Administration Bureau, Wenchuan 623006, China; 13348986271@163.com

**Keywords:** *Salvia przewalskii*, seed germination, seed quality, seed dormancy, pre-sowing treatment, soil substrate

## Abstract

*Salvia przewalskii* Maxim. is a perennial alpine plant with significant ornamental and medicinal value. However, previous studies have shown that this species has a low germination rate under natural conditions, and its artificial propagation techniques remain unclear. This study aimed to investigate the seed quality and the effects of various pre-sowing treatments (storage, chemical, physical, hormonal, combined) and soil substrates on *S. przewalskii* germination. The results indicated that *S. przewalskii* seeds demonstrated high viability (>85%) but exhibited high empty seed rate (45.32%) and physiological dormancy. Compared with the control (20%), dehydration (24.44%), demucilage + dehydration combination (35.56%), storage at 4 °C for 360 (53.33%) and 450 (54.29%) days, and GA_3_ (44.44–55.56%) treatments significantly enhanced *S. przewalskii* germination percentages. Demucilage, H_2_SO_4_ and KNO_3_ treatments had negative effects on seed germination, while 6-BA treatment did not significantly improve seed germination. Among tested soil substrates, *S. przewalskii* seeds pre-chilled for 450 days showed the highest germination rate (71.11%) and optimal seedling growth in peat:vermiculite (3:1), representing the most suitable soil substrate. These findings demonstrate that understanding germination characteristics of *S. przewalskii* is crucial for developing protocols to enhance germination efficiency that can improve large-scale propagation capacity through shorter germination periods, ultimately enhancing the species regeneration potential and protecting its stability in nature.

## 1. Introduction

With more than 1000 species, sage is the largest genus in the Lamiaceae [1]. Because of its various plant and flower types, diverse flower colors, and high aromatic substances, the species are widely used in the horticultural, food and health, cosmetic, and medical industries [2,3,4,5]. There are over 100 species of East Asian *Salvia*, of which more than 80% are distributed in China [6,7]. About 40 of these species, including *Salvia miltiorrhiza* Bge. and *Salvia przewalskii* Maxim., are traditional Chinese medicines that have been widely used since the Eastern Han Dynasty for the treatment of cardiovascular diseases, hypertension, cirrhosis, stroke, and atherosclerosis [8,9,10]. Our preliminary investigation indicates that wild medicinal sage plant resources have declined sharply in recent years due to indiscriminate harvesting by humans [11]. Therefore, studying sage seed germination is of great significance for large-scale production and to meet market demand for medicinal purposes.

*S. przewalskii* is a perennial herb with purple flowers, mainly distributed in the alpine areas of southwest China, and has high ornamental and medicinal value [12,13,14,15]. Therefore, the expanded propagation of *S. przewalskii* is one of the effective ways to address the demand for and supply of medicinal sage. Wu et al. [16] investigated the germination rate of 20 alpine plants, including *S. przewalskii*, and found that its germination rate after 1 month of layer treatment was only 35.47%, indicating that it required less light for germination. Bilinska et al. [15] studied the effect of different storage temperatures on seed germination of *S. przewalskii* and found that storage at 4 °C and 16 °C for one year increased seed germination. These results are important for us to further explore the effect of different pre-sowing treatments on seed germination of *S. przewalskii*.

Mechanical scarification, hormonal treatments, chemical treatments and water treatments have been widely used to improve germination in plant species [17,18,19]. For example, Nazir et al. [19] found that the germination rate of *S. rosmarinus* was significantly increased to 10.00–36.66%, 6.66–24.83%, 10.17–48.33%, and 41.67–59.33%, respectively, compared to control through physical, chemical, hormonal, and pre-chilling and hormonal combined treatments. Dos Santos et al. [20] treated *Euterpe edulis* seeds from three habitats (including lowland, submontane, and montane forest) with water and found that the germination rate was higher in all low water potential treatments than in flooded treatments, and that the germination rate of seeds from the montane forest was the highest among all treatments at −1.0 MPa. In addition, the selection of soil substrates significantly affects seed germination and seedling growth, with suitable substrates providing optimal water supply for seed germination [21,22]. *S. rosmarinus* germinated significantly higher in soil:sand (1:1) than other substrates combinations, and performed the worst in soil:nanozim (1:1) [19]. Therefore, improving seed germination rates through these pre-sowing treatments and soil substrates could alleviate pressure on wild populations and is a viable approach to promote conservation and sustainability.

In this study, we compared seed size, weight, empty seed rate and viability of *S. przewalskii*, and improved the germination rate through storage, chemical, physical, hormonal, and combined (pre-chilling and physical, hormonal) treatments. We explored the suitable soil substrates for germination and growth of *S. przewalskii* through controlled component ratios. Our research aimed to answer the following questions: (1) What is the quality and germination of *S. przewalskii* seeds? (2) Why is the germination rate of *S. przewalskii* seeds low? How to improve the germination rate of *S. przewalskii*? (3) What is the most suitable soil substrate ratio for the germination and growth of *S. przewalskii*? We expect to identify the causes of poor germination in *S. przewalskii*, improve seed germination rates through pre-sowing treatments and soil substrates selection, and provide theoretical guidance for the large-scale production and market application of *S. przewalskii*.

## 2. Results

### 2.1. Seed Quality

**Figure 1:**
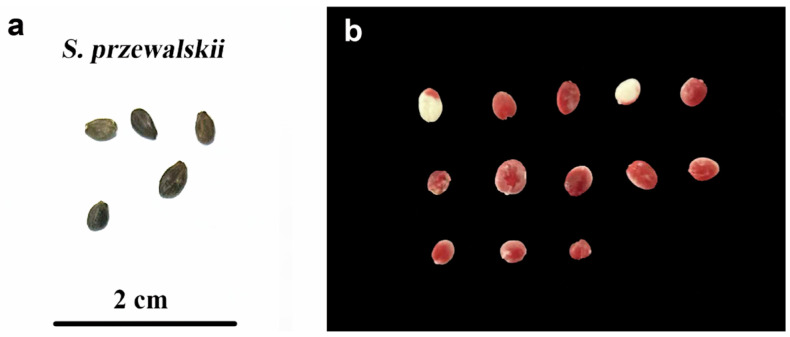
Seed morphology and viability of *Salvia przewalskii*. (**a**) Seed morphology; (**b**) seed viability; red color for viable seeds and white color for nonviable seeds.

The seeds of *S. przewalskii* were long-ovate and tan (Figure 1a). Morphological measurements revealed that the length, width, and thickness of *S. przewalskii* seed were 3.80 ± 0.09 mm, 2.51 ± 0.07 mm, and 1.49 ± 0.03 mm, respectively. The hundred-seed weight of *S. przewalskii* was 0.50 ± 0.00 g, and the empty seed rate was 45.32 ± 0.72%. Notably, *S. przewalskii* seeds exhibited high seed viability, at 87.02 ± 1.26% (Figure 1b).

### 2.2. Germination Response to Storage Times

**Figure 2:**
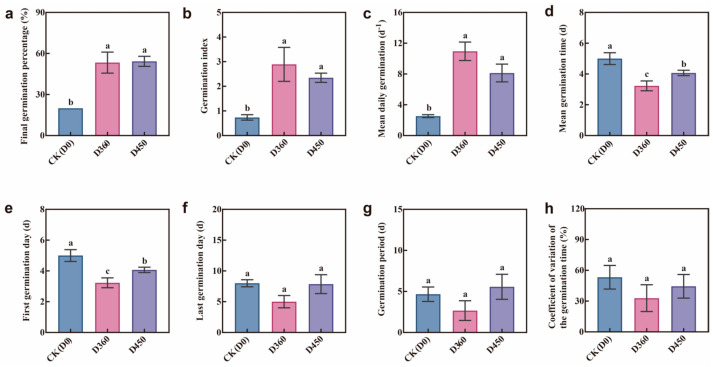
Effect of pre-chilling treatments on germination characteristics of *Salvia przewalskii* seeds. (**a**) Final germination percentage (FGP); (**b**) germination index (GI); (**c**) mean daily germination (MDG); (**d**) mean germination time (MGT); (**e**) first germination day (FGD); (**f**) last germination day (LGD); (**g**) germination period (GP); (**h**) coefficient of variation in the germination time (CVt) (mean ± SEM). CK (D0) refers to fresh seeds. D360 and D450 refers to pre-chilled seeds at 4 °C for 360 and 450 days, respectively. Different lowercase letters indicate significant differences among the treatments (*p* < 0.05).

Both fresh seeds (D0 treatment) and stored seeds (D360 and D450 treatments) of *S. przewalskii* started to germinate within 2–4 days, and the germination percentage of D0 treatment was 20.00 ± 0.00%, significantly lower than that of D360 treatment (53.33 ± 7.70%) and D450 treatment (54.29 ± 3.69%) (*χ*^2^ = 37.438, *dƒ* = 2/10, *p* < 0.001; Figure 2a). Similarly, there was no significant difference in the GI and MDG of *S. przewalskii* in D360 treatment compared to D450 treatment, but both were significantly higher than those of D0 treatment (*χ*^2^ = 23.284, *dƒ* = 2/10, *p* < 0.001; Figure 2b; *χ*^2^ = 22.819, *dƒ* = 2/10, *p* < 0.001; Figure 2c). Comparison of MGT revealed a decreasing and then increasing trend among *S. przewalskii* seeds of the three storage times (*χ*^2^ = 22.346, *dƒ* = 2/10, *p* < 0.001; Figure 2d). The MGT of D0 treatment was the longest, at 5.00 ± 0.38 d, significantly higher than those stored for 360 and 450 days. The MGT of D360 treatment was shortest, at 3.23 ± 0.32 d, significantly lower than those stored for 450 days. However, there was no significant difference in GP and CVt for all three storage times (*χ*^2^ = 2.075, *dƒ* = 2/10, *p* = 0.354; Figure 2g; *χ*^2^ = 1.111, *dƒ* = 2/10, *p* = 0.574; Figure 2h). These results suggest that dormancy exists in fresh seeds of *S. przewalskii*, and pre-chilling treatment can effectively break seed dormancy and shorten the mean germination time.

### 2.3. Germination Response to Chemical Treatments

**Figure 3:**
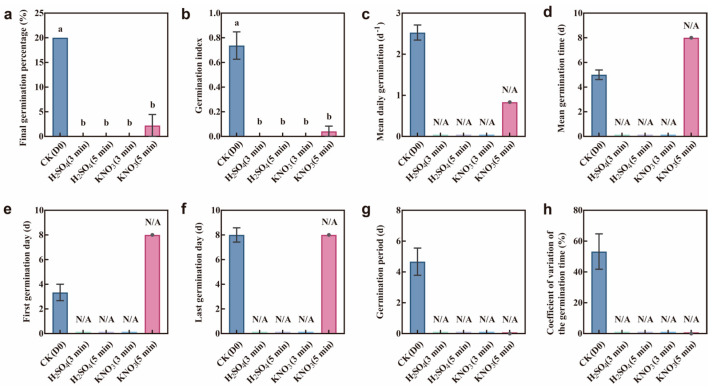
Effect of chemical treatments on germination characteristics of *Salvia przewalskii* seeds. (**a**) Final germination percentage (FGP); (**b**) germination index (GI); (**c**) mean daily germination (MDG); (**d**) mean germination time (MGT); (**e**) first germination day (FGD); (**f**) last germination day (LGD); (**g**) germination period (GP); (**h**) coefficient of variation in the germination time (CVt) (mean ± SEM). CK (D0) refers to fresh seeds. N/A indicates that the data is not applicable due to insufficient germination. Different lowercase letters indicate significant differences among the treatments (*p* < 0.05).

The results of the two chemical treatments, potassium nitrate and sulfuric acid, showed that only one seed in *S. przewalskii* treated with 0.6% potassium nitrate for 5 min germinated on the eighth day, with a germination percentage of 2.22 ± 2.22%, while the rest did not germinate, significantly lower than that of the control by 20.00 ± 0.00% (*χ*^2^ = 465.000, *dƒ* = 4/10, *p* < 0.001; Figure 3a). GI also showed the same trend (*χ*^2^ = 226.909, *dƒ* = 4/10, *p* < 0.001; Figure 3b). These results indicate that the two chemical treatments had a negative effect on seed germination in *S. przewalskii*.

### 2.4. Germination Response to Physical Treatments

**Figure 4:**
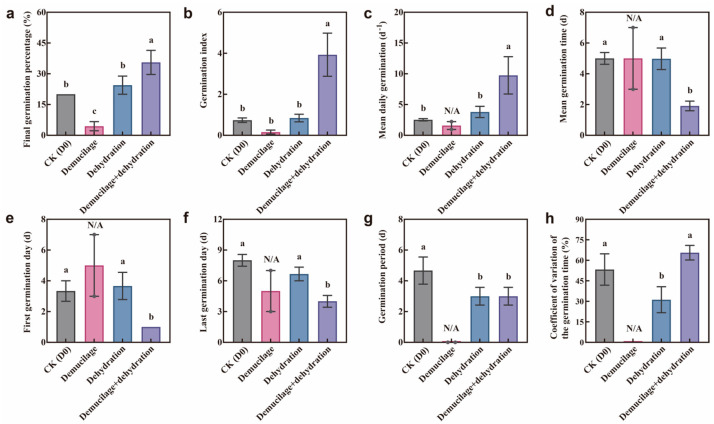
Effect of physical treatments on germination characteristics of *Salvia przewalskii* seeds. (**a**) Final germination percentage (FGP); (**b**) germination index (GI); (**c**) mean daily germination (MDG); (**d**) mean germination time (MGT); (**e**) first germination day (FGD); (**f**) last germination day (LGD); (**g**) germination period (GP); (**h**) coefficient of variation in the germination time (CVt) (mean ± SEM). CK (D0) refers to fresh seeds. N/A indicates that the data is not applicable due to insufficient germination. Different lowercase letters indicate significant differences among the treatments (*p* < 0.05).

The germination percentages of the three physical treatments of demucilage, dehydration and their combination differed significantly (*χ*^2^ = 50.500, *dƒ* = 3/8, *p* < 0.001; Figure 4a). Demucilage treatment significantly reduced *S. przewalskii* seed germination by only 4.44 ± 2.22% compared to the control. The germination rate of dehydration treatment, on the other hand, was not significantly different from the control. The combined treatment of demucilage and dehydration resulted in a germination percentage of up to 35.56 ± 5.88%, which was significantly higher than the control. Similarly, the combined treatment of demucilage and dehydration had the highest GI and MDG of 3.93 ± 1.05 and 4.98 ± 0.07, respectively, which were significantly higher than the control and the other two physical treatments, and there was no significant difference between the dehydration treatment and the control (*χ*^2^ = 47.295, *dƒ* = 3/8, *p* < 0.001; Figure 4b; *χ*^2^ = 38.643, *dƒ* = 2/6, *p* < 0.001; Figure 4c). The combined treatment of demucilage and dehydration concentrated germination on days 1–5, significantly faster than the control and the other two physical treatments (days 3–9) (*χ*^2^ = 15.545, *dƒ* = 2/6, *p* < 0.001; Figure 4e; *χ*^2^ = 33.600, *dƒ* = 2/6, *p* < 0.001; Figure 4f). MGT also showed a consistent trend (*χ*^2^ = 38.643, *dƒ* = 2/6, *p* < 0.001; Figure 4d). CVt comparison revealed no significant difference between the combined treatment of demucilage and dehydration and the control, but both were significantly higher than the dehydration treatment (*χ*^2^ = 10.921, *dƒ* = 2/6, *p* = 0.040; Figure 4h).

### 2.5. Germination Response to Hormonal Treatments

**Figure 5:**
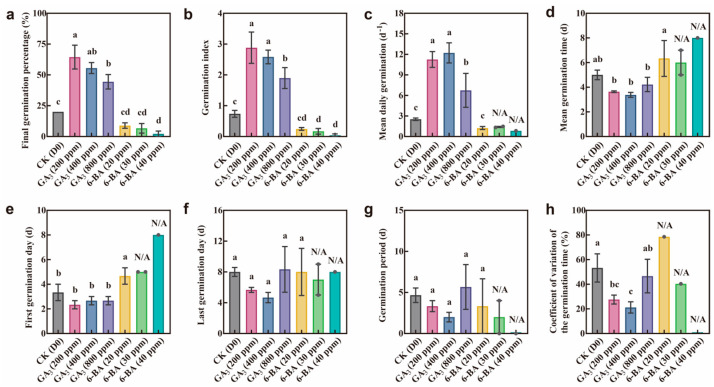
Effect of hormone pre-soaking treatments on germination characteristics of *Salvia przewalskii* seeds. (**a**) Final germination percentage (FGP); (**b**) germination index (GI); (**c**) mean daily germination (MDG); (**d**) mean germination time (MGT); (**e**) first germination day (FGD); (**f**) last germination day (LGD); (**g**) germination period (GP); (**h**) coefficient of variation in the germination time (CVt) (mean ± SEM). CK (D0) refers to fresh seeds. N/A indicates that the data is not applicable due to insufficient germination. Different lowercase letters indicate significant differences among the treatments (*p* < 0.05).

The differences in germination percentages of *S. przewalskii* between the two hormonal treatments, GA_3_ and 6-BA, were significant (*χ*^2^ = 237.000, *dƒ* = 6/14, *p* < 0.001; Figure 5a). Germination percentages decreased progressively with the increase of 6-BA concentration compared to control. The germination percentages of *S. przewalskii* seeds in 20 ppm and 30 ppm 6-BA treatments were slightly lower than those in CK and slightly higher than those in 40 ppm 6-BA treatment, but the differences were not statistically significant. The germination percentage in 40 ppm 6-BA treatment was significantly lower than that in CK. All GA_3_ treatments increased *S. przewalskii* seed germination. The FGP of 200 ppm GA_3_ treatment was the highest (64.44 ± 9.69%), slightly higher than the 400 ppm GA_3_ treatment (55.56 ± 4.44%), but the difference was not significant; the 800 ppm GA_3_ treatment had the lowest germination (44.44 ± 5.88%), significantly lower than the 200 ppm, but not significantly different from the 400 ppm. GI and MDG showed similar trends, with the difference being that the 400 ppm GA_3_ treatment had a significantly higher GI and MDG than those of 800 ppm GA_3_ treatment, and the GI of 30 ppm 6-BA treatment was significantly lower than that of the control (*χ*^2^ = 222.099, *dƒ* = 6/14, *p* < 0.001; Figure 5b; *χ*^2^ = 76.035, *dƒ* = 4/10, *p* < 0.001; Figure 5c). The FGD of the three GA_3_ treatments was slightly earlier than the control, but there was no significant difference and all of them were significantly earlier than the 20 ppm 6-BA treatment (*χ*^2^ = 21.273, *dƒ* = 4/10, *p* = 0.363; Figure 5e). MGT also showed a similar trend, but the difference between the 20 ppm 6-BA treatment and the control was not significant (*χ*^2^ = 16.147, *dƒ* = 4/10, *p* = 0.003; Figure 5d). Comparing LDG and GP, it was found that there was no significant difference between the three GA_3_ treatments and 20 ppm 6-BA treatment and the control (*χ*^2^ = 4.333, *dƒ* = 4/10, *p* = 0.363; Figure 5f; *χ*^2^ = 2.950, *dƒ* = 4/10, *p* = 0.566; Figure 5g). The CVt of the 400 ppm GA_3_ hormonal treatment was the lowest at 21.20 ± 4.59%, significantly lower than 800 ppm GA_3_ treatment (46.66 ± 13.61%) and the control (53.26 ± 11.47%), but not significantly different from 200 ppm GA_3_ treatment (27.58 ± 3.62%) (*χ*^2^ = 11.893, *dƒ* = 3/8, *p* = 0.008; Figure 5h).

### 2.6. Germination Response to Pre-Chilling Combination Treatments

**Figure 6:**
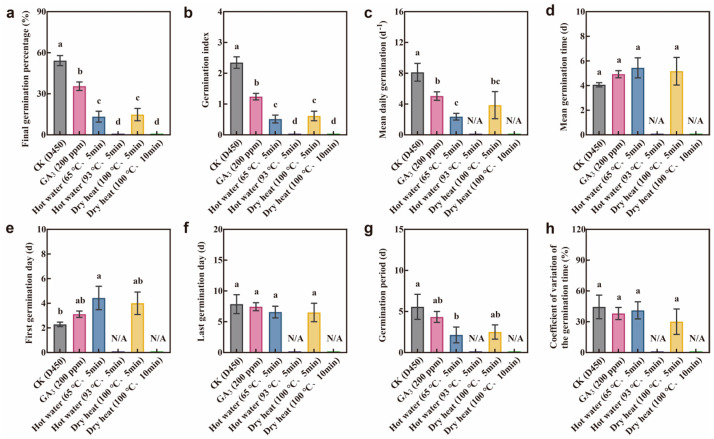
Effect of recombination treatments on germination characteristics of *Salvia przewalskii* seeds. (**a**) Final germination percentage (FGP); (**b**) germination index (GI); (**c**) mean daily germination (MDG); (**d**) mean germination time (MGT); (**e**) first germination day (FGD); (**f**) last germination day (LGD); (**g**) germination period (GP); (**h**) coefficient of variation in the germination time (CVt) (mean ± SEM). CK (D450) refers to pre-chilled seeds at 4 °C for 450 days. N/A indicates that the data is not applicable due to insufficient germination. Different lowercase letters indicate significant differences among the treatments (*p* < 0.05).

All pre-chilling + physical and pre-chilling + hormonal combination treatments had significantly lower germination percentages than the control (D450 treatment). In the pre-chilling + hot water bath treatments, the seed germination percentages were significantly decreased to 0 with the increase in the water bath temperature. And in the pre-chilling + dry heat treatments, the seed germination percentages were also significantly reduced to 0 with the increase in the dry heat time. Among them, the germination percentage of the pre-chilling + dry heat (100 °C, 5 min) combination treatment (14.79 ± 4.51%) was slightly higher than that of the pre-chilling + hot water (65 °C, 5 min) combination treatment (13.33 ± 3.98%), but there was no significant difference. Both of them were significantly higher than the pre-chilling + hot water (93 °C, 5 min) and pre-chilling + dry heat (100 °C, 10 min) combination treatment (both at 0.00 ± 0.00%), and significantly lower than pre-chilling + 200 ppm GA_3_ combination treatment (35.56 ± 3.14%) (*χ*^2^ = 328.654, *dƒ* = 5/41, *p* < 0.001; Figure 6a). GI also showed a consistent trend (*χ*^2^ = 389.458, *dƒ* = 5/41, *p* < 0.001; Figure 6b). Similarly, the MDG of pre-chilling + dry heat (100 °C, 5 min) was slightly lower than that of the control and pre-chilling + 200 ppm GA_3_ combination treatment, and slightly higher than that of the pre-chilling + hot water (65 °C, 5 min) combination treatment, but there was no significant difference. Whereas the MDG of the pre-chilling + 200 ppm GA_3_ combination treatment was not significantly different from the control, it was significantly higher than the pre-chilling + hot water (65 °C, 5 min) combination treatment (*χ*^2^ = 27.024, *dƒ* = 3/23, *p* < 0.001; Figure 6c). Compared with the control, there were no significant differences in MGT, LGD, and CVt for all the pre-chilling combination treatments (*χ*^2^ = 3.866, *dƒ* = 3/23, *p* = 0.276; Figure 6d; *χ*^2^ = 1.154, *dƒ* = 3/23, *p* = 0.764; Figure 6f; *χ*^2^ = 1.250, *dƒ* = 3/20, *p* = 0.741; Figure 6h). Except for the GP being significantly lower than the control in the pre-chilling + hot water (65 °C, 5 min) combination treatments, there were no significant differences between the other combination treatments and the control (*χ*^2^ = 7.679, *dƒ* = 3/23, *p* = 0.053; Figure 6g).

### 2.7. The Influence of Soil Substrates on Germination and Seedling Growth

**Figure 7:**
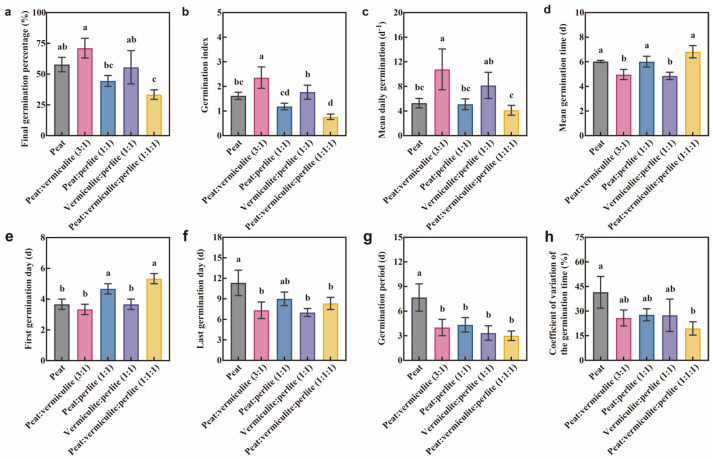
Effect of different potting media combinations on germination characteristics of *Salvia przewalskii* seeds. (**a**) Final germination percentage (FGP); (**b**) germination index (GI); (**c**) mean daily germination (MDG); (**d**) mean germination time (MGT); (**e**) first germination day (FGD); (**f**) last germination day (LGD); (**g**) germination period (GP); (**h**) coefficient of variation in the germination time (CVt) (mean ± SEM). Different lowercase letters indicate significant differences among the treatments (*p* < 0.05).

Significant differences were observed between the germination percentages of different types of soil substrates and ratios (Figure 7a). The highest germination percentage of *S. przewalskii* seeds was found in peat:vermiculite (3:1) (71.11 ± 8.01%), significantly higher than peat:perlite (1:1) (44.44 ± 4.44%) and peat:vermiculite:perlite (1:1:1) (33.33 ± 3.85%), and slightly higher than peat (57.78 ± 5.88%) and perlite:vermiculite (1:1) (55.56 ± 13.52%), but the difference was not significant (*χ*^2^ = 19.359, *dƒ* = 4/10, *p* < 0.001; Figure 7a). GI also showed a similar trend (*χ*^2^ = 33.718, *dƒ* = 4/10, *p* < 0.001; Figure 7b). Similarly, the MDG of *S. przewalskii* seeds in peat:vermiculite (3:1) was the highest, which was slightly higher than perlite:vermiculite (1:1), but there was no significant difference, significantly higher than the other three groups (*χ*^2^ = 12.838, *dƒ* = 4/10, *p* = 0.012; Figure 7c). Perlite:vermiculite (1:1) had the lowest MGT, at 4.86 ± 0.30 d, slightly lower than peat:vermiculite (3:1), but not significantly different, and all were significantly lower than the remaining three groups (*χ*^2^ = 28.648, *dƒ* = 4/10, *p* < 0.001; Figure 7d). A similar trend was observed for FGD and LGD, with the earliest initiation of germination on the 3rd to 4th day in peat, peat:vermiculite (3:1) and perlite:vermiculite (1:1), which differed significantly from the other two groups. Peat:vermiculite (3:1), perlite:vermiculite (1:1) and peat:vermiculite:perlite (1:1:1) ended germination as early as at 5–10 days, significantly earlier than peat, but not significantly different from peat:perlite (1:1) (*χ*^2^ = 37.800, *dƒ* = 4/10, *p* < 0.001; Figure 7e; *χ*^2^ = 12.714, *dƒ* = 4/10, *p* = 0.013; Figure 7f). However, the peat treatment had the longest GP and CVt, with a GP of 7.67 ± 1.67 d, significantly higher than the remaining four groups, and a CVt of 41.52 ± 16.73%, significantly higher than peat:vermiculite:perlite (1:1:1), with no significant difference with the other three groups (*χ*^2^ = 18.412, *dƒ* = 4/10, *p* = 0.001; Figure 7g; *χ*^2^ = 7.989, *dƒ* = 4/10, *p* = 0.092; Figure 7h). These results indicate that peat:vermiculite (3:1) had the highest germination percentage and germination index, shorter mean germination time, more concentrated germination period, and was the most suitable for seed germination of *S. przewalskii*.

**Figure 8:**
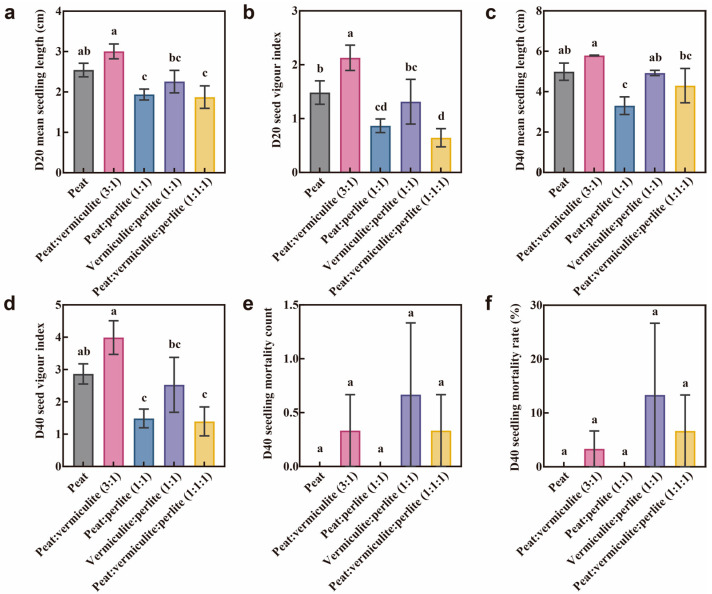
Effect of combination of potting media on growth characteristics of *Salvia przewalskii* seeds. (**a**) D20 mean seedling length (Ls); (**b**) D20 seed vigor index (SVI); (**c**) D40 mean seedling length (Ls); (**d**) D40 seed vigor index (SVI); (**e**) D40 seedling mortality count; (**f**) D40 seedling mortality rate (mean ± SEM). Different lowercase letters indicate significant differences among the treatments (*p* < 0.05).

**Figure 9:**
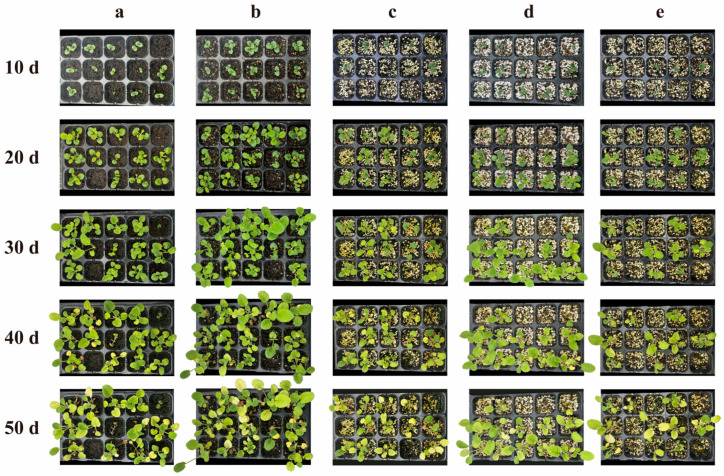
Germination and seedling growth of *Salvia przewalskii* seeds pre-chilled for 450 days in different combinations of potting media for 10–50 days. (**a**) Peat; (**b**) peat: vermiculite (3:1); (**c**) peat: perlite (1:1); (**d**) vermiculite:perlite (1:1); (**e**) peat: vermiculite: perlite (1:1:1).

There were significant differences in the mean seedling length (Ls) and seedling vigor index (SVI) of *S. przewalskii* seedlings grown in different soil substrates (Figure 8 and Figure 9). The highest Ls of 3.01 ± 0.18 cm was observed in peat:vermiculite (3:1) on the 20th day, which was slightly higher than peat but not significantly different, and significantly higher than the other three groups; the lowest Ls of 1.87 ± 0.28 cm was observed in peat:vermiculite:perlite (1:1:1), which was slightly lower than peat:perlite (1:1) and perlite:vermiculite (1:1), but the difference was not significant (*χ*^2^ = 27.731, *dƒ* = 4/10, *p* < 0.001; Figure 8a). Similarly, the SVI of peat:vermiculite (3:1) on the 20th day was the highest at 2.13 ± 0.24, significantly higher than the other four groups, and the SVI of perlite:vermiculite (1:1) was significantly higher than peat:vermiculite:perlite (1:1:1), slightly higher than peat:perlite (1:1), and slightly lower than peat, but not significantly different (*χ*^2^ = 31.454, *dƒ* = 4/10, *p* < 0.001; Figure 8b).

On the 40th day, Ls and SVI showed a similar pattern, with peat:vermiculite (3:1) still performing the best of the five groups, with a significantly higher Ls (5.79 ± 0.02 cm) than peat:perlite (1:1) (3.31 ± 0.44 cm) and peat:vermiculite:perlite (1:1:1) (4.30 ± 0.85 cm) (*χ*^2^ = 23.025, *dƒ* = 4/10, *p* < 0.001; Figure 8c). And its SVI (3.99 ± 0.52) was significantly higher than the other four groups (1.40–2.86) (*χ*^2^ = 25.050, *dƒ* = 4/10, *p* < 0.001; Figure 8d). With the increase in time, there were seedlings that gradually died, but there was no significant difference in D40 seedling mortality count (*χ*^2^ = 3.500, *dƒ* = 4/10, *p* = 0.478; Figure 8e) and mortality rate (*χ*^2^ = 4.000, *dƒ* = 4/10, *p* = 0.406; Figure 8f). Specifically, one plant died in the peat:vermiculite (3:1) treatment, two in perlite:vermiculite (1:1), and one in peat:vermiculite:perlite (1:1:1). No seedling death was observed in the other treatments. These results indicate that peat:vermiculite (3:1) had the highest mean seedling length and seedling vigor index among the five groups on day 20 and 40, and had no significant difference in seedling mortality rate compared to the other four groups, making it the most suitable for the seedling growth of *S. przewalskii*.

## 3. Discussion

Seeds are the basis for plant growth and development, as well as the formation of the next generation of offspring. They are one of the most important aspects of plant reproductive strategies and factors such as seed size, hundred-seed weight, viability and empty rate are prerequisites for studying seed developmental processes, and may affect water absorption and germination of seeds [23,24,25]. In this study, *S. przewalskii* seeds had a higher empty seed rate at 45.32%, with seed viability as high as 85%, but the germination percentage of fresh seeds was only 20.00%, which suggested that *S. przewalskii* seeds are of low quality and are also dormant. *S. przewalskii* grows in the alpine region of southwest China, where harsh climatic and environmental conditions, infertile soils, and habitat fragmentation allow for impeded gene exchange and homogenization, leading to a decline in population genetic diversity. Furthermore, successive visits by pollinators to flowers on the same plant may promote inbreeding, leading to insufficient pollination and ultimately reducing seed quality. It has also been found that *S. przewalskii* shares the pollinator, *Bombus trifasciatus*, with *Delphinium yuanum*, a sympatric coflowering species, and the heterospecific pollen transfer of *S. przewalskii* is higher than that of *D. yuanum*, resulting in lower reproductive success [26]. These may be three of the reasons why this species is endangered under natural conditions. Therefore, understanding the factors affecting the germination of *S. przewalskii* and exploring the optimal germination conditions are crucial for its artificial propagation and effective conservation.

Application of chemical methods to induce seed germination and break dormancy is a common method to increase seed percentage. Sulfuric acid immersion is one of the most commonly used chemical methods. Concentrated sulfuric acid can scratch dormant seeds through corrosion, effectively break seed dormancy and significantly promote seed germination [27,28]. Potassium nitrate can not only induce the synthesis of growth hormone in plants and regulate their vigor, but also in eliminating the effect of germination inhibiting substances on seeds, so that seed viability is improved and germination is promoted [29,30,31]. However, in this study, the germination percentage of seeds treated with concentrated sulfuric acid and potassium nitrate immersion ranged from 0% to 2.22%, both of which were significantly lower than that of CK, and failed to play the role of breaking dormancy and promoting germination. Our findings are in agreement with those of Phuyal et al. [18], who found that the germination percentage of *Zanthoxylum armatum* seeds soaked in concentrated sulfuric acid for 1, 2, and 5 min failed to show significant differences compared to the control. And treating the seeds with different acids for a long time will damage the seed coat and prevented the seeds from germinating at all, which may be related to the damage to the embryos [32]. Previous studies have found that the presence of mucilage significantly increases sage seed germination under various environmental conditions [33]. In this study, we also observed similar results that the germination rate of *S. przewalskii* seeds in demucilage treatment was significantly lower than that of the control. Furthermore, *S. przewalskii* seeds treated with potassium sulfate and potassium nitrate also exhibited lower germination rates, which may be due to the chemical agents disrupting the seed mucilage during soaking, thereby reducing germination rates. In future research, the influence of chemical methods on the germination of *S. przewalskii* should be further explored by reducing the concentration of chemicals and soaking time.

Physical treatment is a common pre-sowing technique that breaks physiological dormancy in many plants. Seed coat mucilage plays important roles in germination, including increasing water content as water storage, facilitating hydration during seed absorption, adhering to the soil or animal bodies to facilitate dispersal, preventing oxygen diffusion, DNA repair, participating in biodegradation to provide nutrients, and protecting the seedling under biotic or abiotic stresses [34,35]. It has been shown that germination of *S. hispanica* seeds with demucilage was slower, with a germination percentage of 63% after 26 h, compared to 97% germination percentage of intact seeds [36]. This is consistent with the results of this study, where we found that the germination percentage of the demucilage treatment was significantly lower than that of CK, and that the dehydration treatment had little effect on breaking dormancy in *S. przewalskii*. This may be related to the fact that seed germination requires a certain amount of water and oxygen as well as the mucilage’s ability to increase surface area, promote water absorption and retention, and impede oxygen acquisition. Moreover, this study found that the combination of the two treatments produced a synergistic effect, which not only effectively broke the seed dormancy of *S. przewalskii* and increased germination percentage, but also resulted in faster germination speed.

Studies have shown that a low degree of seed dormancy is widely present in the Lamiaceae [36,37], and 6-BA and GA_3_ are two hormones often used to break seed dormancy. In this study, GA_3_ hormonal treatment significantly increased the germination percentage (44.44–64.44%) and slightly decreased MGT, which is consistent with the results reported by *S. rosmarinus* and *S. officinalis* [20,38]. 6-BA is a plant growth regulator with the functions of promoting cell proliferation and inducing flower bud differentiation. Through soaking treatment, the hormone level in the plants can be change and increase the content of growth substances in plants, which in turn improves plant growth and development [39]. In contrast, 6-BA hormonal treatment had no significant effect on seed germination. This may be related to the induction of metabolites or enzymes that inhibit seed germination by 6-BA.

In recent years, it has been shown that low-temperature treatment can increase the germination percentage of dormant seeds by reducing ABA levels on the embryonic axis, decreasing protein phosphatase gene expression and regulating ABA/GA_3_ balance [40,41,42]. In this study, *S. przewalskii* seeds pre-chilled for 360 and 450 days exhibited significant germination percentages compared to non-pre-chilled seeds. FGP, GI and MDG did not change significantly with increasing pre-chilling time, but MGT increased significantly, indicating that the germination ability was similar in both groups of pre-chilling treatments, but prolonged pre-chilling time slowed down the germination speed of seeds. This is consistent with the findings of Bilinska et al. [16] that the total number of germinated seeds (*G*_max_), germination energy and germination capacity increased significantly in *S. przewalskii* after one year of storage at 4 °C and 16 °C. We also carried out a combination of pre-chilling with physical and hormonal treatments; however, the germination percentages of all the combination treatments were significantly lower than those of the pre-chilled seeds. CVt, LGD, and MGT showed not significant difference compared to pre-chilled seeds, indicating that the combination of physical and hormonal treatments could not further break the dormancy of the pre-chilled seeds, nor could they improve germination percentage or accelerate the germination process. Pre-chilling treatment may have maximized the release of dormancy in *S. przewalskii* seeds, and the combination of pre-chilling treatment may have triggered thermal damage or hormonal inhibitory effects, leading to lower germination percentage.

Soil substrates can provide the required moisture and nutrients for seed germination, and different seeds have their relatively suitable substrates environment. A study has found that *S. rosmarinus* prefers a loose, breathable soil substrate, and its germination percentage and seedling vigor index were highest in soil:sand (1:1), significantly higher than other soil substrates [20]. In this study, we found that *S. przewalskii* germinated and grew best in the peat:vermiculite (3:1), with the highest germination percentage, mean seedling length, and seedling vigor index among the five groups. However, the peat:vermiculite:perlite (1:1:1) treatment performed the worst, suggesting that *S. przewalskii* not only requires a soil substrate with certain degree of permeability, but also needs to grow in a nutrient rich, water and fertilizer retaining substrate. This is related to the growth habits of *S. przewalskii,* which grows in alpine soils with low organic matter content and is afraid of drought and flooding. It may also be related to the fact that peat–vermiculite treatment can more effectively increase soil organic carbon content, optimize soil rhizosphere microbial communities and increase crop yields [43].

## 4. Materials and Methods

### 4.1. Experimental Materials

*S. przewalskii* seeds were collected in 2023 and 2024 in Baoxing County, Ya’an City, China (102°45′ E, 30°40′ N, 2500 m above sea level). After air-drying in shade (seed moisture ≈ 10%), the seeds were placed in kraft paper bags and stored in air tight containers. The 2023 seeds were stored at 4 °C for 360 and 450 days, while the 2024 seeds were stored at room temperature.

### 4.2. Seed Size, Weight, Empty Seed Rate and Viability

To investigate seed size, weight, empty seed rate, and viability, we thoroughly mixed *S. przewalskii* seeds collected in 2023 and 2024 and performed the following treatments: (1) 10 seeds were randomly selected, and their length, weight and thickness were measured with a vernier caliper (Series 530, Mitutoyo America Corporation, Aurora, IL, USA). (2) Using the hundred-seed method to measure seed weight, 300 seeds were randomly selected (100 seeds per replicate) and weighed using a electronic analytical scale (ME204, Mettler Toledo Inc., Columbus, OH, USA). (3) Using the soaking method to determine empty seed rate, 300 seeds were randomly selected (100 seeds per replicate) and soaked for 24 h. Floating seeds were considered empty seeds. (4) In total, 60 seeds were randomly selected (20 seeds per replicate) to determine seed viability. The seed coats were removed, and the stripped embryos were stained with 1% TTC in a thermostat at 30 °C protected from light for 24 h. Embryos stained red indicated viability. The specific calculation formulas are shown in Table 1.

### 4.3. Effects of Storage Times on Germination

To investigate whether dormancy exists in fresh seeds of *S. przewalskii* and the effect of storage on breaking dormancy, we used fresh seeds as a control and conducted pre-chilling treatment, which refers to testing the germination of seeds after 360 and 450 days of storage at 4 °C. We randomly selected 45 seeds (15 seeds per replicate) to detect the presence of dormancy in fresh seeds. Subsequently, 45 seeds stored for 360 days (3 replicates) and 90 seeds stored for 450 days (7 replicates) were randomly selected for germination. The seeds were then sown in sterilized Petri dishes (90 mm diameter) lined with two layers of filter paper and containing 10–15 mL of ultrapure water. Based on the environmental conditions and the inhibition of light on the germination of *S. przewalskii* in Wu et al. [17], the Petri dishes were wrapped in tinfoil and placed in a dark 26–28 °C artificial climate incubator (RDN-1000D-4, Ningbo Southeast Instrument Co., Ltd., Ningbo, China) in the seed germination experiment. In the soil substrates experiment, we set the artificial climate incubator to a light intensity of 80 µmol photons M^−2^S^−1^ with 16 h of light and 8 h of darkness. Seed germination was observed daily after sowing, with the length of radicle over 2 mm considered indicative of germination. The experiment lasted for 20 days.

### 4.4. Chemical Treatment

To examine the effect of chemical treatments on the germination of *S. przewalskii*, we used untreated fresh seeds as a control and immersed the fresh seeds in 60% sulfuric acid and 0.6% potassium nitrate for 3 and 5 min, respectively. Three replicates of 15 seeds were used for each treatment. Temperature and light conditions were the same as in Section 4.3.

### 4.5. Physical Treatments

To examine the effect of physical treatments on the germination of *S. przewalskii*, we used untreated fresh seeds as a control and employed sunk high-quality fresh seeds that have just been soaked for 24 h to the following three physical treatments: (1) Demucilage treatment, which refers to gently wiping off the mucilage on the surface of the seeds with filter paper. (2) Dehydration treatment, which refers to dispersing the seeds on sulphate paper and drying them in a dry and ventilated place for 24 h. (3) The combined treatment of demucilage and dehydration refers to removing the mucilage from the seed coat and then drying the seeds for 24 h. Three replicates of 15 seeds were used for each treatment. Temperature and light conditions were the same as in Section 4.3.

### 4.6. Hormonal Treatments

To examine the effect of hormone treatments on the germination of *S. przewalskii*, we used untreated fresh seeds as a control and soaked the fresh seeds in gibberellic acid (GA_3_) (200, 400, and 800 ppm) and 6-benzyladenine (6-BA) (20, 30, and 40 ppm) for 24 h, respectively. Three replicates of 15 seeds were used for each treatment. Temperature and light conditions were the same as in Section 4.3.

### 4.7. Combination of Pre-Chilling and Physical, Hormonal Treatments

The experiment aimed to evaluate the impact of pre-chilling treatment on seed germination in combination with physical and hormonal treatments. Using *S. przewalskii* seeds pre-chilled at 4 °C for 450 days as the control, we subjected the pre-chilled seeds described above to the following five treatments: (1) Soaking in hot water at 65 °C for 5 min (8 replicates); (2) soaking in hot water at 93 °C for 5 min (12 replicates); (3) dry heat at 100 °C for 5 min (4 replicates); (4) dry heat at 100 °C for 10 min (5 replicates); and (5) soaking in 200 ppm GA_3_ for 24 h (9 replicates), with 15 seeds per replicate. Temperature and light conditions were the same as in Section 4.3.

### 4.8. Effects of Soil Substrates on Germination and Seedling Growth

To investigate the effects of different soil substrates on seed germination and seedling growth of *S. przewalskii*, we used the seeds pre-chilled at 4 °C for 450 days as experimental material, tested various ratios of peat, vermiculite and perlite, and set up the following five treatments: (1) Peat; (2) peat:vermiculite (3:1, *v*/*v*); (3) peat:perlite (1:1, *v*/*v*); (4) vermiculite:perlite (1:1, *v*/*v*); and (5) peat:vermiculite:perlite (1:1:1, *v*/*v*). Three replicates of 15 seeds were used for each treatment. Temperature and light conditions were the same as in Section 4.3. Subsequently, when the seedlings grew to the 20th and 40th day, we measured the mean seedling length (Ls) of all seedlings, calculated the seed vigor index (SVI), and seedling mortality count and rate on the 40th day (Table 1).

### 4.9. Statistical Analysis

In this study, the following parameters were calculated with reference to the methods of Nazir et al. [20] and Ismaili et al. [44]: final germination percentage (FGP), germination index (GI), mean daily germination (MDG), mean germination time (MGT), germination period (GP), and coefficient of variation of the germination time (CVt). The specific calculation formulas are shown in Table 1.

All statistical analyses were performed using IBM SPSS Statistics 27 (Chicago, IL, USA). All figures were created using GraphPad Prism 10.2.1 (Boston, MA, USA). Differences in seed size, weight, emptying rate and viability between the two species were analyzed using a generalized linear model (GLM) with the species as independent variables and different seed quality parameters (including length, width, size index, weight, emptying rate, and viability) as dependent variables. We also assessed the effects of storage, chemical, physical, hormonal, combination of pre-chilling and physical, and combination of pre-chilling and hormonal treatments on seed germination of *S. przewalskii* using GLM separately with the treatments as independent variables and various germination parameters (including FGP, GI, MDG, MGT, FGD, LGD, GP, CVt, and SVI) as dependent variables.

## 5. Conclusions

This study evaluated the seed quality of *S. przewalskii*, investigated the effects of different pre-sowing treatments on seed germination of *S. przewalskii*, explored the optimal pre-sowing treatment and soil substrate for *S. przewalskii*, and answered three main research questions. The results of this study indicated that *S. przewalskii* has higher seed viability, but is hindered by seed dormancy, resulting in a low germination percentage and making reproduction under natural conditions difficult. Physical, hormonal and storage treatments can induce the germination process with 200 ppm and 400 ppm GA_3_ treatments and 360 and 450 days storage treatments being the most effective, followed by 800 ppm GA3 treatment. However, chemical treatments cannot break seed dormancy, and the combination treatments of demucilage and dehydration is superior to pre-chilling and hormonal treatments in breaking dormancy and accomplishing germination in a shorter period of time. Pre-chilling does not present synergistic effects with physical and hormonal combined treatment. In terms of soil substrates, peat:vermiculite (3:1) is the most effective ratio to ensure survival while enhancing seedling growth, which can be used to achieve maximum germination and seedling growth for nursery growth in *S. przewalskii*.

Therefore, establishing a standardized germination protocol for the species is crucial for its large-scale reproduction and material use in the pharmaceutical industry. In this study, we improved seed germination percentage by optimizing the seed treatment protocol for *S. przewalskii*, thus providing an effective method for its cultivation in both natural habitats and artificial environments. The developed reproducible protocol will reduce the dependence on plant material from natural habitats and promote the sustainable use of this plant species.

## Figures and Tables

**Table 1:** The method of calculating seed germination and seedling growth.

Studied Indices and Calculation Formula
Seed Size Index = (Length + Width + Thickness)/3
Empty Seed Rate = (number of floating seeds/total number of observed seeds) × 100
Seed Viability = embryos stained red/total number of embryos
Final Germination Percentage (FGP) = (*N*g/*N*t) × 100
Germination Index (GI) = ∑(*G*t/*D*t)
Mean Daily Germination (MDG) = FGP/D
Mean Germination Time (MGT) = ∑(*ni* × *di*)/*N*t
Germination Period (GP) = LGD − FGD
Coefficient of Variation of the Germination Time (CVt) = (*s_t_*/MGT) × 100
Seed Vigor Index (SVI) = (Ls × FGP)/100
Seedling Mortality Rate = (Seedling mortality count/Total number of seedlings) × 100

*N*g = number of germinated seeds; *N*t = total number of seeds planted; *G*t = number of germinated seeds on the tth day; *D*t = number of corresponding germination days; D = experiment period; *ni* = germinated seeds per day; *di* = counting day; LGD = last germination day; FGD = first germination day; *s_t_* = standard deviation of the germination time; Ls = mean seedling length.

## Data Availability

The data presented in this study are available on request from the corresponding author. The data are not publicly available due to privacy and ethical restrictions.
